# Heat Sink Effect on Tumor Ablation Characteristics as Observed in Monopolar Radiofrequency, Bipolar Radiofrequency, and Microwave, Using Ex Vivo Calf Liver Model

**DOI:** 10.1097/MD.0000000000000580

**Published:** 2015-03-06

**Authors:** Krishna Pillai, Javid Akhter, Terence C. Chua, Mena Shehata, Nayef Alzahrani, Issan Al-Alem, David L. Morris

**Affiliations:** From the Department of Surgery, University of New South Wales, St. George Hospital, Kogarah, New South Wales, Australia.

## Abstract

Thermal ablation of liver tumors near large blood vessels is affected by the cooling effect of blood flow, leading to incomplete ablation. Hence, we conducted a comparative investigation of heat sink effect in monopolar (MP) and bipolar (BP) radiofrequency ablation (RFA), and microwave (MW) ablation devices.

With a perfused calf liver, the ablative performances (volume, mass, density, dimensions), with and without heat sink, were measured. Heat sink was present when the ablative tip of the probes were 8.0 mm close to a major hepatic vein and absent when >30 mm away. Temperatures (T1 and T2) on either side of the hepatic vein near the tip of the probes, heating probe temperature (T3), outlet perfusate temperature (T4), and ablation time were monitored.

With or without heat sink, BP radiofrequency ablated a larger volume and mass, compared with MP RFA or MW ablation, with latter device producing the highest density of tissue ablated. MW ablation produced an ellipsoidal shape while radiofrequency devices produced spheres.

Percentage heat sink effect in Bipolar radiofrequency : Mono-polar radiofrequency : Microwave was (Volume) 33:41:22; (mass) 23:56:34; (density) 9.0:26:18; and (relative elipscity) 5.8:12.9:1.3, indicating that BP and MW devices were less affected.

Percentage heat sink effect on time (minutes) to reach maximum temperature (W) = 13.28:9.2:29.8; time at maximum temperature (X) is 87:66:16.66; temperature difference (Y) between the thermal probes (T3) and the temperature (T1 + T2)/2 on either side of the hepatic vessel was 100:87:20; and temperature difference between the (T1 + T2)/2 and temperature of outlet circulating solution (T4), Z was 20.33:30.23:37.5.

MW and BP radiofrequencies were less affected by heat sink while MP RFA was the most affected. With a single ablation, BP radiofrequency ablated a larger volume and mass regardless of heat sink.

## INTRODUCTION

Thermal ablation with radiofrequency waves is a widely used technique for treating both primary and secondary malignant hepatic tumors.^1,2^ Recently, microwave (MW) thermal ablation has been proposed to have several advantages over radiofrequency ablation (RFA) of liver tumors.^3,4^ The primary goal of thermal ablation of tumor is to ensure complete destruction of tumors that are dependent on creating a safety margin of 10 mm around the external border of the tumor.^5,6^ However, the success of thermal ablation is dependent on several factors such as tumor size, location, hepatic blood flow, techniques, and equipment selection.^7,8^ Notably, the proximity of tumor to hepatic blood flow has been shown to influence the success rate of complete tumor eradication.^9–12^ Other studies have also indicated that the size of ablation is regulated by the proximity of perfusion in hepatic tissues.^13,14^ Hence, hepatic blood flow with its cooling properties affecting tumor ablation is commonly termed “heat sink effect.” Many researchers attribute tumor recurrence, after ablation, to the heat sink phenomena.

At present, 2 different types of RFA devices are clinically in use. The monopolar (MP) RFA uses a single antenna while the bipolar (BP) RFA utilizes dual antennas. The RFA was the first commercially available device and is commonly used for liver tumor ablation owing to ease of application. However, the more recent BP has been shown to be capable of ablating a much larger volume of tumor compared with MP, in a single ablation^15^ and at the same time, is least affected by heat sink compared with MP.^16^ Recently, microwave (MW) ablation device with also a single antenna has been introduced. Researchers have suggested that MW has several advantages over the conventional RFA devices, notably superior heating capacity, absence of charring effect, and with minimal heat sink effect.^17,18^

Hence, in this study, we investigate 3 ablation technologies (MP, BP, and MW) in order to compare their ablation efficacy in the presence and absence of heat sink. In order to do this, we monitored the ablation parameters of mass, volume, lateral and longitudinal dimensions, along with temperature profile during ablation. For liver model, a continuously perfused ex vivo calf liver was used, with relative heat sink effect as a function of distance from major hepatic blood vessel (as determined by ultrasound guidance). The distance of 30 mm from the major blood vessel was utilized as the spacing at which heat sink was fully attenuated.^19–21^

## MATERIALS AND METHODS

No ethics approval was required to perform this study as it does not involve the use of patient tissues. Calf liver from the abattoir was perfused with 2% heparin solution to clear all the blood clots in the blood vessels. The liver was temporarily stored in crushed ice before experimental use and was equilibrated to 37°C before beginning the experiment. Two liters of Ringer solution was used as perfusate. The 2 RFA probes used in the experiment were RFA (Rita Starbust-2) manufactured by (RITA, Mountain View, CA) while the in circle BP probe, type and size (03-48425), 5 × 25 cm (length), was from (Medical Inc, Fremont, CA); MW antenna (Medwaves-gauge 14) was supplied by MedWaves Incorporated, San Diego, CA, and the ultrasound device used in the experiment was ALOKA S&D-650CL supplied by Hitachi Aloka America, Wallingford, CT. The 3 thermal probes were used according to the manufacturer's protocol to ablate a volume with lateral diameter of 5.0 cm.

**FIGURE 1:**
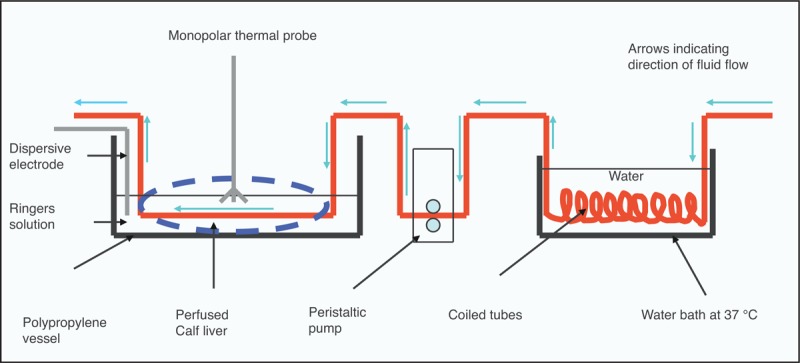
General set-up for performing the experiment using monopolar (MP) radiofrequency ablation device. The set-up was similar for microwave (MW) ablation and for bipolar (BP) radiofrequencyablation devices, 2 antennas were used that pierced the liver parallel to each other. In the case of BP and MW, there was no dispersive electrode.

The flow set-up of the experiment was arranged as shown in Figure 1 for MP, a similar set-up as for MW but without the dispersive probe, and 2 probes for BP. The liver perfusate (Ringer solution) was circulated through a coiled tube that was immersed in a water bath kept constant at 37°C, at a flow rate of 500 mL/min.

**FIGURE 2:**
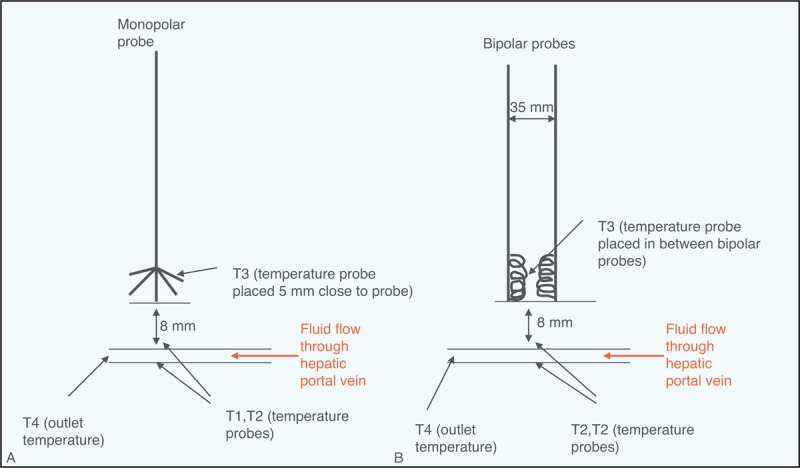
(A) Strategic placement of temperature sensors T1 and T2 for monopolar (MP) radiofrequency ablation alongside the hepatic portal vein. (B) Similar arrangement carried out with bipolar (BP) radiofrequency ablation. For microwave, a similar arrangement as for MP was carried out except temperature sensor T3 that was not required because it was already monitored by the generator device with continuous recording. The placement of temperature sensors T3 for both the MP and the BP are also shown Figure 2A and B.

**FIGURE 3:**
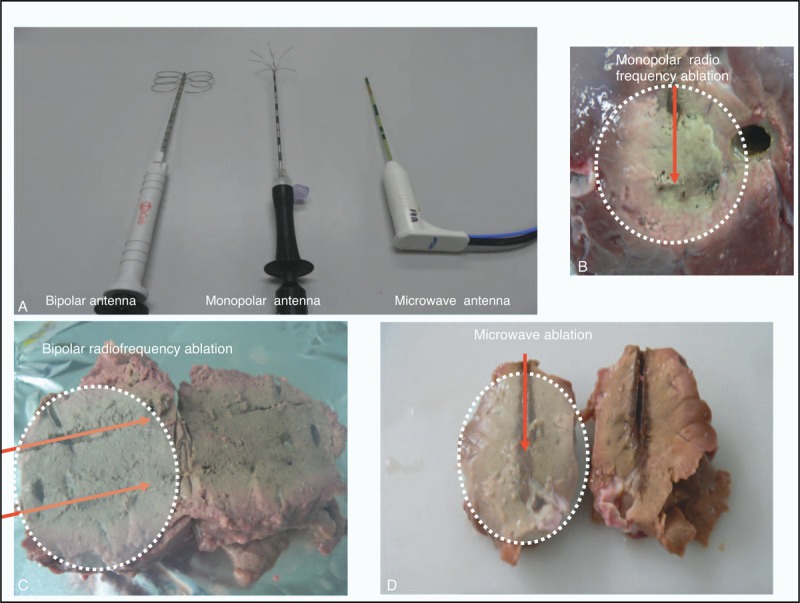
(A) Three types of ablation devices, monopolar and bipolar radiofrequency (2 identical probes but only 1 is displayed) and microwave, that have been used in this experiment. (B)–(D) Ablated tissues (longitudinal section) using monopolar radiofrequency, bipolar radiofrequency, and microwave devices, respectively. Both monopolar and bipolar radiofrequency ablation produce a more spherical shape compared with microwave. The red arrows denote the position of the antennas in the tissue.

With ultrasound guidance, temperature probes, thermocouples (T1, T2), were strategically placed on either side of the blood vessel, vertically below the antenna. For vessel simulation, the blood vessel was located 8 mm below the probes (Figure 2A and B). For RFA, another temperature probe (T3) was placed 5 mm close to the needles of the probe. For BP probes, T3 was placed in between the probes at the coiled end, but close to one of them at 5.0 mm. In the case of MW, the temperature of the probe was automatically monitored by the generator and recorded as T3. The outflow of the solution from perfusion was monitored using a temperature probe T4. The temperatures for both MP and BP were continuously monitored with the help of reader and printer. The 3 thermal probes used for ablation are shown in Figure 3A.

The volume of liver tissue that was ablated (recognized by the change in color from deep brownish red to grayish pink) was measured by the water displacement method by immersing only the tissue ablated into the water. The tissue ablated was surgically excised from the rest of the liver. The lateral and longitudinal dimensions of ablated tissues were measured using a linear centimeter scale while the mass ablated were also recorded in grams. For each device, the experiment was repeated 3 times and means readings with standard deviations were recorded.

The power generator set-ups for the devices along with time are as follows:

1. MP RFA—110 watts; ablation time, 20 minutes
2. BP RFA—110 watts; ablation time, 10 minutes
3. MW ablation—25–28 watts (as recommended by the manufacturer for the probe); temperature 110°C; ablation time, 17 minutes

## RESULTS

### Comparison of Ablation Parameters Between BP, MP, and MW

#### Heat Sink Absent

The mean volume, mass, density, and elipscity of the 3 ablations and their comparative ratios are shown in Table 1. The volume and mass ablated follow a trend with BP having the highest value followed by MW and with the lowest value in MP. The density of ablated tissues with MW was slightly higher than that for BP and considerably higher compared with MP. The density of tissues indicated how well the tissues were ablated with each device The elipscity of tissues indicates how close to spherical shape the ablation were, the order of closeness to a spherical shape being BP, MP, and MW. MW was ellipsoidal while BP and MP were more or less spherical (Figure 3B–D).

**TABLE 1:**
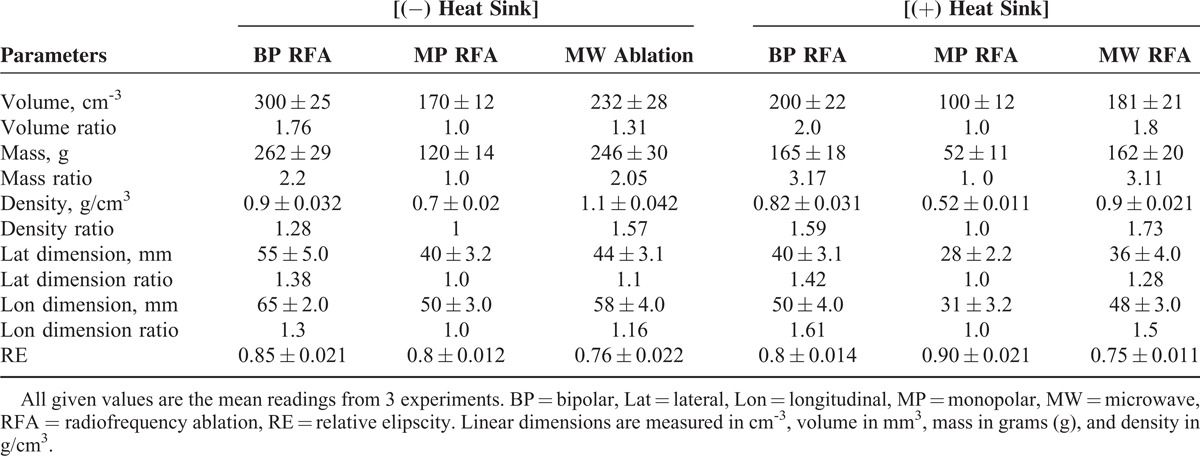
Comparison of Tumor-Ablated Parameters Using MP, BP, and MW Thermal Devices With (+) and Without (−) Heat Sink in Perfused Calf Liver

All the 3 devices were set to ablate a lateral dimension of 50 mm; however, their performance indicates that BP exceeded the limit by 10% (55–50/50), MP fell short by 20% (50–40/50), and MW also fell short by 12% (50–44/50).

#### Heat Sink Present

The mean volume, mass, density, and elipscity of the 3 ablations and their comparative ratios are shown in Table 1. The volume ablated is highest in BP, followed by MW, and lowest in MP, and a similar trend for the mass ablated.

The density of ablated tissue was highest in MW with BP being slightly smaller. However, the density of tissues ablated in MP was considerably smaller compared with both MW and BP. The elipscity shows that for both BP and MP, the shape of ablated tissue was close to being a sphere; however, for MW, it was again ellipsoidal.

### Comparison of Ablation Parameters, in Presence to Absence of Heat Sink

**FIGURE 4:**
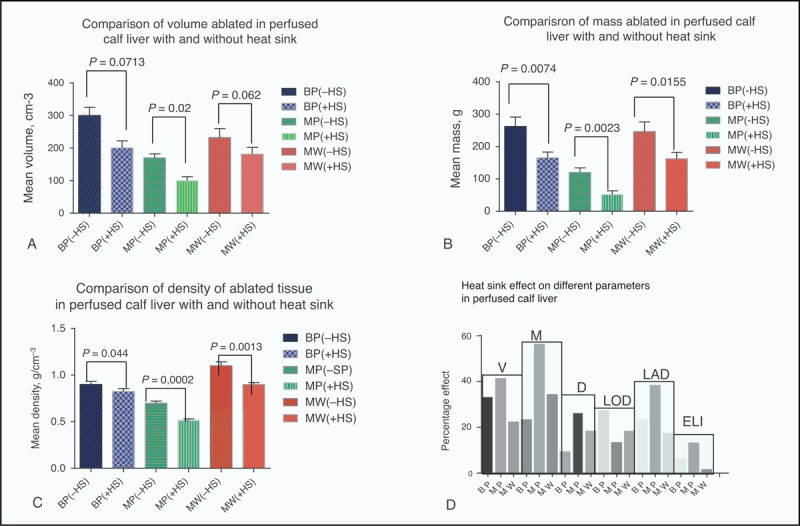
(A)–(C) Comparison of liver tumor ablative parameters that were measured in the absence and presence of heat sink. (D) Graphical representation of the percentage heat sink effect on measured ablative parameters. BP = bipolar, D = density, ELI = elipscity, -HS = heat sink absent, +HS = heat sink present, LAD = lateral dimension, LOD = longitudinal dimension, M = mass, MP = monopolar, MW = microwave, V = volume

Comparing the mean volumes ablated (Table 2, Figure 4A–D), MP was most affected (41%), MW was least affected (22%), and BP with 33% was slightly more affected compared with MW. For effect of heat sink on mean mass, BP was least affected (23%) with MW being slightly more affected (34%) while MP was most affected (56%). The mean density measurements show that BP was least affected (9%), while MW (18%) was affected almost twice as compared with BP and MP with 26% being most affected. Volume, mass, and density, in the presence to the absence of heat sink in all the 3 devices, were statistically significant (*P* = < 0.05). Comparing the effect of heat sink on mean elipscity, MW was least affected (1.3%) while BP with a value of 5.8% was slightly more affected and MP with 12.9% was most affected.

**TABLE 2:**
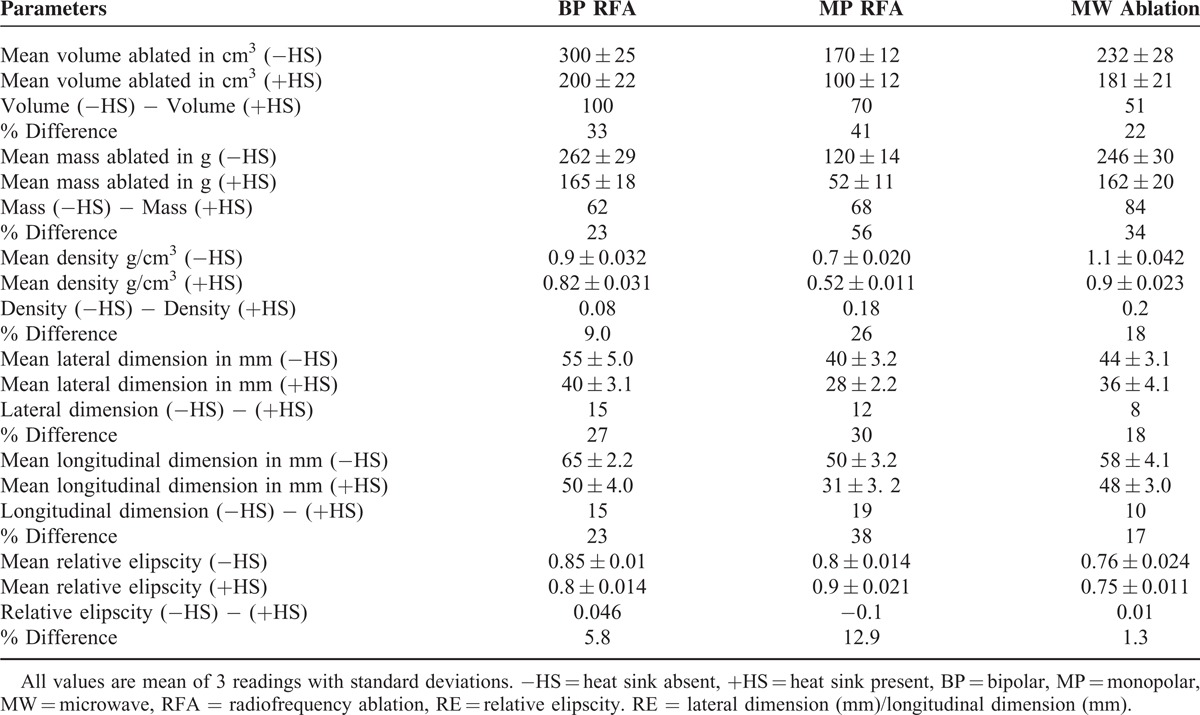
Comparison of Heat Sink Effect as Observed in BP RFA, MP RFA, and MW Ablation

### Analysis of Heat Sink Effect on Time and Temperature Profile During Ablation

**FIGURE 5:**
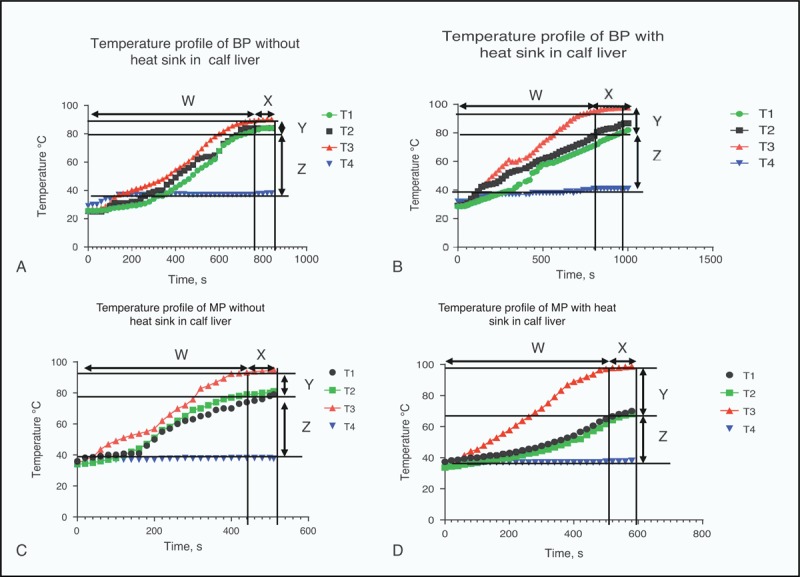
Temperature profile with time, (A) and (B) for BP in perfused liver and (C) and (D) for MP RFA. BP = bipolar, MP = monopolar, RFA = radiofrequency ablation, W = time in minutes to reach maximum temperature, X = time at which maximum temperature was maintained, Y = temperature difference between (T1 + T2)/2, and T3, T1, and T2 are temperatures on either side of the perfused hepatic portal vein, respectively, and T3 is the temperature of the ablative thermal probe, Z = temperature difference between (T1 + T2)/2 and outlet circulating fluid temperature T4. Tmax = maximum temperature reached during ablation.

**FIGURE 6:**
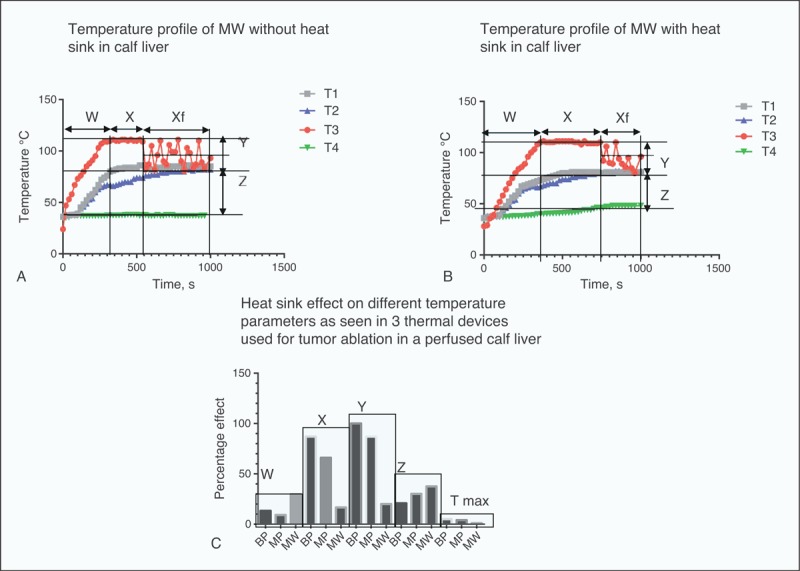
Temperature profile with time, (A) and (B) for MW ablation of perfused liver while (C) shows a comparative heat sink effect on various time and temperature profile in BP RFA, MP RFA, and MW devices. BP = bipolar, MP = monopolar, MW = microwave, RFA = radiofrequency ablation, W = time in minutes to reach maximum temperature, X = time at which maximum temperature was maintained, Y = temperature difference between (T1 + T2)/2 and T3, T1, and T2 are temperatures on either side of the perfused hepatic portal vein, and T3 is the temperature of the ablative thermal probe, Z = temperature difference between (T1 + T2)/2 and outlet circulating fluid temperature T4, Tmax = maximum temperature reached during ablation, Xf = time at which maximum temperature fluctuates.

The mean duration of time to reach maximum ablation temperature (W) was significantly affected in MW by the presence of heat sink (29.8%) as compared with either BP (13.28%) or MP (9.2%), as shown in Table 3. However, the mean duration of time during which maximum temperature was maintained during ablation (X) was least affected in MW (16.6%) as compared with large differences in both BP (87%) and MP (66%), Figures 5 and 6.

**TABLE 3:**
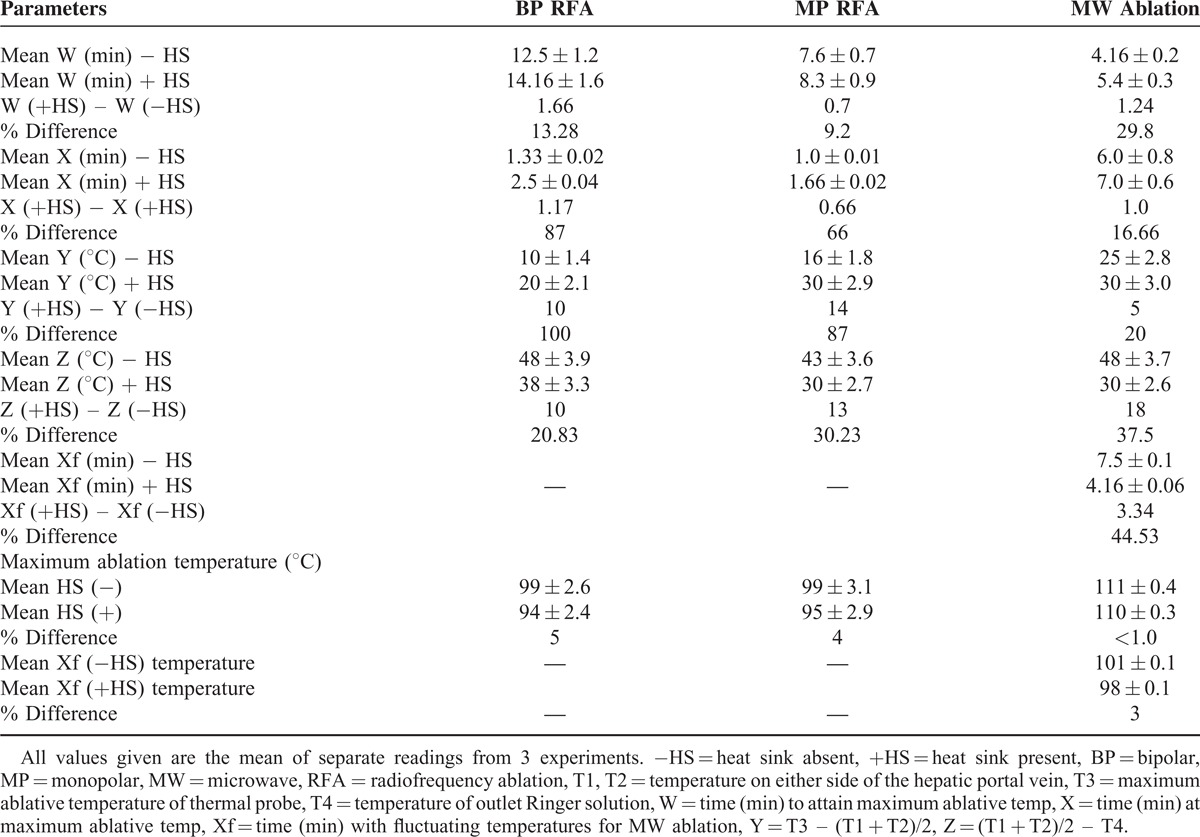
Analysis of Time and Temperature Profile From Beginning to End of Ablation

The mean temperature difference between the thermal device (T3) and temperatures of T1 and T2 placed below the thermal probes on either side of the hepatic portal vein was indicative of how close the 3 temperatures were and was related to heat sink. This was denoted by Y and it appears that the percentage difference that was affected by heat sink was comparatively small in MW (20%) as compared with MP (87%) and BP (100%). This indicates that the heat sink effect affecting temperatures of ablation tissues near major blood vessel was small in MW compared with the 2 radiofrequency devices.

The mean temperature difference between (T1 + T2)/2 and T4 (temperature of outlet hepatic portal circulating fluid as indicated by Z) shows that BP has the smallest value of (20.8%) as compared with MW of 37.5% and MP of 30.23%. This may indicate the amount of heat lost through circulating fluids as indicated by T4. Hence, the percentage difference in Z value when heat sink was present would indicate the amount of heat lost, the smaller, the less heat was lost. Therefore, MW has the highest heat loss second to MP while BP has minimal heat loss. The difference between MW and BP is 16.7% indicating that heat loss due to heat sink in MW is almost twice that by BP.

The Xf value was the time period at which the maximum ablation temperature drops and fluctuates in MW device. This was only observed in MW ablation since it was set on temperature mode (maximum of 111°C) and hence during the Xf period, it fluctuated and maintained a mean value of 101°C without heat sink and 98°C, in the presence of heat sink. Analysis shows that heat sink dramatically affected this period of temperature fluctuation by 44.53%. Also the mean fluctuating temperature in the Xf zone was affected by 3% with heat sink.

Finally, when the mean maximum ablation temperature in the 3 devices was examined for heat sink effect (X zone), it appears that MW was least affected (<1.0%) while it was 5% in BP and 4% in MP. However, owing to the fluctuation of ablation temperature in the Xf zone, MW was only able to maintain its maximum temperature to an average of 101°C without heat sink and 98°C, in the presence of heat sink.

## DISCUSSION

Although ablative parameters for the 3 thermal devices (MP, BP, and MW) were preset, to ablate a spherical shape of diameter 5.0 cm (according to manufacturer's recommendation), the volumes and mass of tissues ablated, regardless of heat sink indicated that BP outperformed the other 2 devices. However, MW showed slightly more dense ablated tissues that may be indicative of the degree of ablative necrosis since volume shrinkage occurs with loss of humidity.^22^ The shapes of ablated tissues were more spherical in the case of BP and MP while it was ellipsoidal for MW. It is conceivable that the ablated shape may have some bearing on the efficacy of ablation on spherical tumors with a single procedure.

Furthermore, the 3 devices did not adhere to the recommended performance, BP outperformed by 10%, while MP and MW fell short by 20% and 12%, respectively. This may be due to variance in tissues and other parameters that may have affected the performances of these devices. In the clinical setting, variations of tissues exist in the liver and may depend on tumor size, density, and the presence of cirrhotic tissues.^23,24^

Heat sink affects all 3 devices to varying degrees. Both volume and mass was most affected in MP (41% and 56%, respectively). A lesser effect was seen in BP (33% and 37%) while in MW, it was 22% and 34%, indicating that the last 2 devices were more comparable. The relative differences in the effect on volume and mass may indicate the efficacy of ablation, as a result of heat sink. This was further confirmed by the densities where MP was most affected (25.7%) and MW was also substantially affected (18.2%), while BP was minimally affected (8.8%). Although the heat sink effect on density of tissue ablated was higher in MW, the overall density of tissues ablated still remained higher in MW (0.9 g/cm^3^) compared with BP of 0.825 g/cm^3^. The capacity to heat surrounding tissues more efficiently by MWs may have contributed to this observation.^25^ Evaluation of heat sink on lateral and longitudinal dimensions ablated also showed lesser effect in MW compared with MP and BP. This also translated to a similar finding on elipscity.

Although thermal ablation of tissues are temperature and time dependent,^26,27^ any fluctuations in these parameters may affect the tissues ablated. The mean time (W) taken to reach maximum ablative temperature in MW was most affected (29.8%) compared with either BP (13.28%) or MP (9.2%). Furthermore, the mean time duration (X) at maximum ablative temperature was most affected in BP (87%) compared with either MP (66%) or MW (16.6%). Although heat sink affected the duration of time to reach maximum ablative temperature the most in MW, it is a clear indication that rapid heating by MW with steep heat gradient set-up results in rapid loss of heat. However, the mean time duration (X) at which maximum ablative temperature was maintained was least affected (16.6%) indicating that once maximum temperature was reached, any loss of heat through heat sink was rapidly compensated. Compared with MW, the time at maximum temperature (X) was noticeably affected in BP (87%) and in MP (66%). The duration of time where maximum ablative temperature was maintained would certainly have an effect on ablation parameters, particularly for RFA devices since it depended very much on conductive heat transfer.^28^ In the case of MW, the time to reach maximum ablative temperature may also affect its ablative performance since ablation period at maximum temperature was shortened. However, in the case of RFA, this extension of time may not affect the performance of the probe as much, since it was dependent on the charring effect with impedence.^29^

The temperature of the thermal probes (T3) were much higher compared with the surrounding tissues on either side of the hepatic portal vein (T1 and T2) that clearly indicated that conduction of heat was nonuniform in all the 3 devices. In the presence of heat sink, the temperature differential was affected in all the 3 devices, BP by a 100%, MP by 87%, while MW with 20% was least affected. This observation may be due to the superior MW heating mechanism (dipole agitation and hysteresis) that was able to compensate heat sink, as compared with electromagnetic heating mechanism in RFA devices. Despite the excess heat sink effect on this temperature differential, the volume and mass of tissue ablated by BP was comparatively larger than either that of MW or MP, suggesting that the parallel heating mode by the 2 probes in BP may have a role in overcoming heat sink effect.

The difference in temperature between the tissues surrounding the hepatic portal vein (T1 + T2/2) and that of the outlet Ringer solution (T4) may also indicate the magnitude of heat loss. The smaller the difference, the greater will be the heat lost. Remarkably, BP had only a 20.83% heat loss while MW with 37.5% had almost twice the heat loss. MP with 30.23% had also a substantial heat loss. This may be due to the parallel heating arrangement between the 2 BP probes, with heating taking place in a parallel fashion between the probes. The MP had a dispersive probe placed at a distance and the current flowed in different directions through the tines from heating probe to complete the electrical circuit. Although the MW had both the poles in a single needle, the rapid heating process generated a steep heat gradient with consequential rapid heat loss through the circulating fluid. The current findings seem to contradict the popular notion that the MW has minimal heat loss through vascular circulation.^17,30,31^ However, MW has an advantage of being able to compensate this heat loss through rapid heating that is characteristic of MW technology.^32^

Presence of heat sinks affects the tissues ablated near the vessels and dependent on the diameter and the flow rate in the vessel.^17^ However, heat sink may also affect the maximum temperature of ablation since heat was conducted from the whole mass of ablation. On a comparative basis, MW reached a maximum temperature of 111°C while the other 2 reached 99°C. Heat sink effect on maximum ablation temperature was very minimal on MW (<1%); however, they were slightly higher in both BP (5%) and MP (4%). At 42°C, cell death was achieved between 3 and 50 hours depending upon tissues types,^33^ and beyond 42°C, there was an exponential decrease in exposure time required for cytodestruction, that is, 8 minutes at 46°C and 2 minutes at 51°C.^34^ At temperatures >60°C, intracellular proteins become denatured, lipid by layer melts, and cell death was inevitable.^35^ Hence, slight fluctuation in temperatures <100°C may not affect the cytodestructive potential of heat.

In the current set-up of the experiment, there may be a slight difference between the temperature of the heating probe (T3) as measured in the experiment, compared with the actual temperature of the probes for both BP and MP, although thermocouples were placed close to the probes. In the case of BP, T3 was placed in between the coiled ends of 2 antennas (15 mm from both the coiled end of antenna) and hence the actual ablative temperature of the probe may be >98°C as recorded. However, for MP, T3 was 5 mm close to the probe and hence represents a temperature that may be closer to the actual antenna temperature. This disparity should not affect the interpretation of our results since we were attempting to record the heat sink effect that is observed in the current set-up of the experiment mainly through ablative parameters of tissues such as volume, mass, density, and elipscity.

The dimensions of the ablated tissues were measured after slicing of the ablated tissues. It was carried out uniformly within 1 mm close to the ablated tissues and hence facilitated the determination of the mass of ablated tissues and subsequently the density. Measuring the lateral and longitudinal dimensions and calculating the volume using a formula^36,37^ was based on the assumption that the ablated tissues were of uniform shape (sphere or ellipse), in reality the ablated tissues do not have a uniform shape (from our experience). This may be due to variations in liver tissues. Furthermore, we opted to examine the temperature profile to study how the heat sink affected these parameters in order to further confirm our findings on heat sink effect on volume mass and density.

Ringer solution that has been chosen for perfusion through the liver may not share similar conductive properties of blood or simulated blood; however, it still demonstrates the principal of heat loss. Furthermore, the perfusion rate in the hepatic portal vein would have a direct bearing on heat loss^38^; however, we choose on a single perfusion speed in order to simplify our model. The heat sink effect by blood flow in the hepatic portal vein may also vary from the hepatic artery owing to differences in diameter, thickness, blood composition, etc,^39^ although we choose to investigate venous circulation as about 70% of blood perfusing the liver is of venous origin.^40^ Hence, in clinical setting, the heat sink effect would depend on a number of factors with hyperemia further increasing the perfusion rate.^38^

Although we did not examine closely, the thermal damage to the hepatic vessels, earlier study had shown that MP causes dramatic damage to tissues far beyond the vessels as compared with BP, indicating that both vessels and other tissues may be damaged during ablation.^16^ This is in agreement with other reports on MP.^41^ On the contrary, MW may appear to cause minimal damage to neighboring large blood vessels, since heat transfer as demonstrated by the temperatures on either side of the blood vessel (T1,T2) and the outlet temperature of circulating fluid (T4), was small in this experiment further indicating that heat is being conducted rapidly away from the blood vessels. As stated earlier, temperatures >60°C causes rapid tissue necrosis and hence despite the rapid conduction of heat in MW, damage to blood vessels is foreseeable. The effect of heat on neighboring blood vessels has also been shown to be dependent on perfusion rate^42^ and the damage may be proportional to perfusion that is dependent on vessel diameter. Hence, vessel damage may vary within the ablation site and from patients to patients based on liver perfusion and the closeness to either smaller or larger blood vessels, the latter possibly with lesser damage.^43^

Indeed, in terms of heat sink effect found in the 3 devices, MW seems to have a comparatively minimal effect and this is agreement to other investigators who have compared MW with MP.^17^ At present, we have compared MW with BP and found that the heat sink effect on volume, mass, and density are quite comparable between the 2 devices. In terms of lateral, longitudinal, and elipscity that was affected by heat sink, MW is once again least affected compared with BP and MP. The heat sink effect on temperature profiles that were examined were also favorable for MW as compared with BP and MP suggesting that MW suffers from least heat sink. However, in terms of volume and mass ablated in a single ablation, BP outperforms the other 2 devices. Although the volume ablated may be increased with overlapping multiple thermal ablation using unipolar devices, it carries several disadvantages such as multiple punctures with an increase in chances of incomplete ablation.^44^

Heat sink remains a major concern for adequate in situ thermal therapy of lesions close to blood vessels, our experiment has shown that while MW was significantly less affected compared with MP, BP appears to have considerable advantage in terms of volume and mass ablated and less affected by heat sink in a perfused liver model compared with MW further suggesting that BP is a practical thermal ablative device. Further verifications in clinical settings are required, although the present findings are rather interesting.
